# Author Correction: Peptidoglycan synthesis drives a single population of septal cell wall synthases during division in *Bacillus subtilis*

**DOI:** 10.1038/s41564-024-01725-7

**Published:** 2024-05-15

**Authors:** Kevin D. Whitley, James Grimshaw, David M. Roberts, Eleni Karinou, Phillip J. Stansfeld, Séamus Holden

**Affiliations:** 1 Centre for Bacterial Cell Biology, Biosciences Institute, Faculty of Medical Sciences, Newcastle University, Newcastle upon Tyne, UK; 2 School of Life Sciences, University of Warwick, Coventry, UK; 3 Department of Chemistry, University of Warwick, Coventry, UK

**Keywords:** Single-molecule biophysics, Bacterial physiology, Bacteriology, Microscopy

Correction to: *Nature Microbiology* 10.1038/s41564-024-01650-9, published online 13 March 2024

In the version of the article initially published, the Data availability section included an incorrect link. This has now been amended in the HTML and PDF versions to include the correct DOI for source data, 10.25405/data.ncl.c.7078312.

