# Supplementary material for: Peptidoglycan synthesis drives a single population of septal cell wall synthases during division in Bacillus subtilis
Source: Nat Microbiol. 2024 Mar 13;9(4):1064–74. doi: 10.1038/s41564-024-01650-9 (PMC10994842; doi:10.1038/s41564-024-01650-9)
Supplement: Supplementary file 1 — Supplementary Figs. 1–12. [file 41564_2024_1650_MOESM1_ESM.pdf]

nature microbiology

Article

https://doi.org/10.1038/s41564-024-01650-9

# Peptidoglycan synthesis drives a single population of septal cell wall synthases during division in *Bacillus subtilis*

In the format provided by the authors and unedited

# SUPPLEMENTARY FIGURES

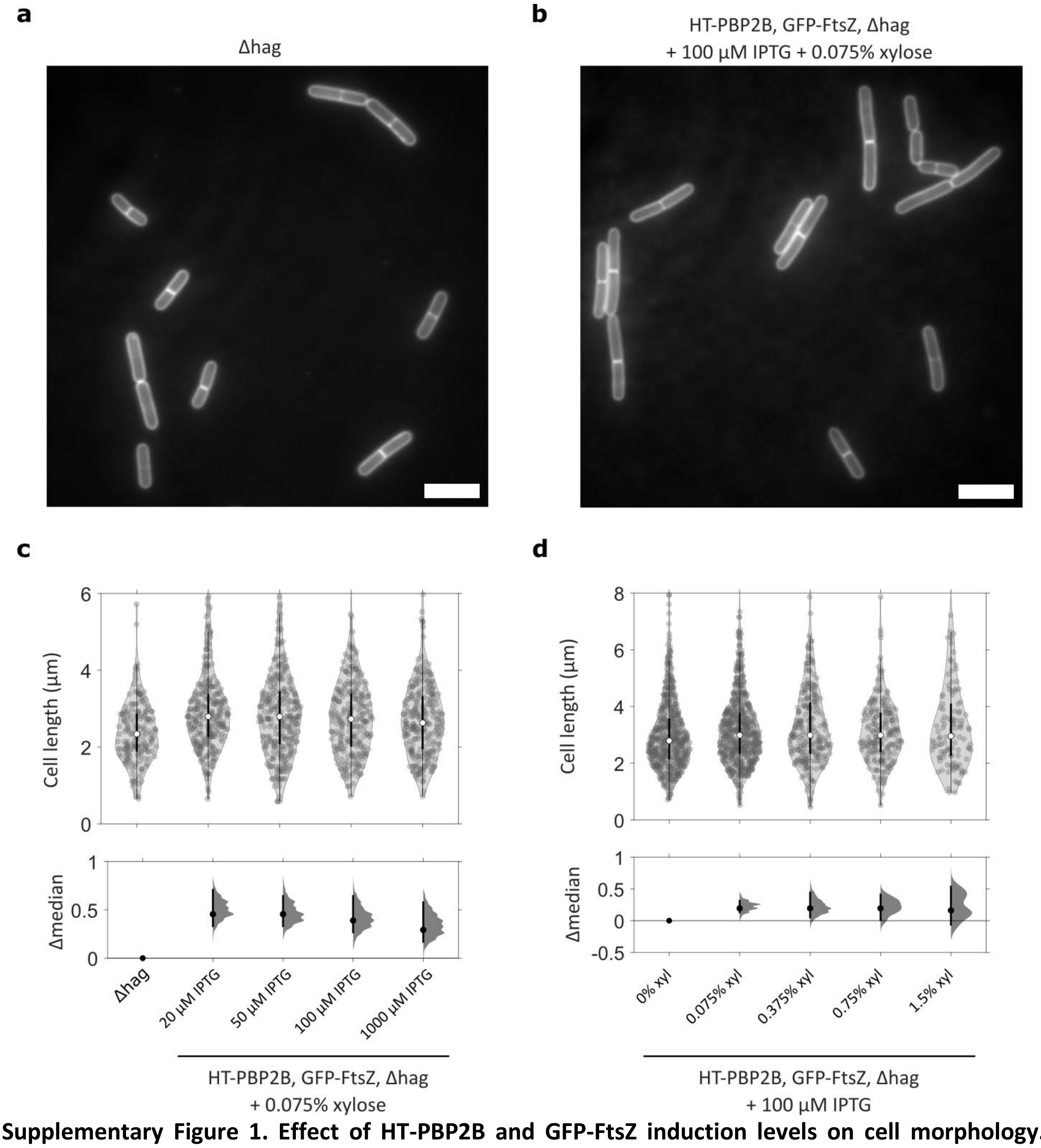


**Supplementary Figure 1. Effect of HT-PBP2B and GFP-FtsZ induction levels on cell morphology.** Lengths of HT-PBP2B GFP-FtsZ Δhag cells (strain SH147) were measured by microscopy of Nile Red-stained cells and compared to those of Δhag cells (strain SH211) (Supplementary Table 1). Both strains were grown in PHMM at 30°C. **(a)** Micrograph of Δhag cells. **(b)** Micrograph of HT-PBP2B, GFP-FtsZ, Δhag cells with 100 µM IPTG and 0.075% xylose (standard experimental conditions). **(c)** HT-PBP2B expression was induced at the level specified by the [IPTG] shown, while GFP-FtsZ levels were held constant (0.075% xylose induction). **(d)** GFP-FtsZ expression was induced at the level specified by the [xyl] concentration shown, while the HT-PBP2B levels were held constant (100 µM IPTG induction). Violin plots: white circles, median; thick black lines, interquartile range; thin black lines, 1.5x interquartile range. DABEST plots: black circle, median difference between indicated conditions; black lines, 95% confidence interval of median difference. Scale bars: 5 µm. Sample sizes are listed in Supplementary Table 6.

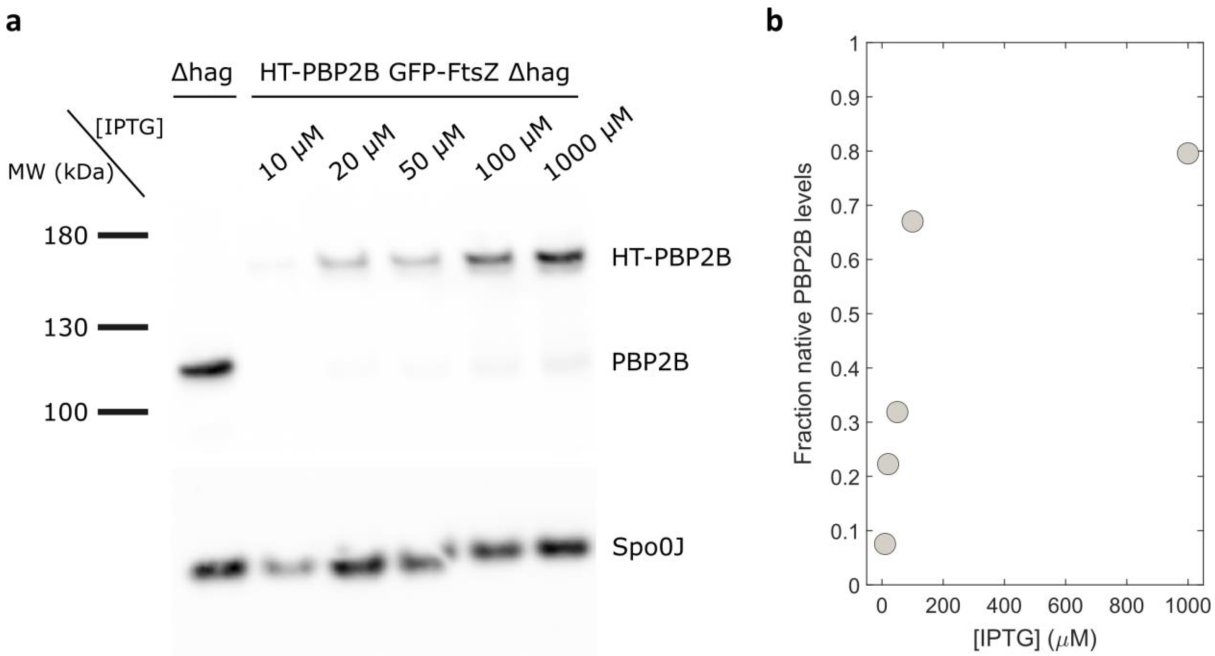


**Supplementary Figure 2. Quantification of PBP2B levels across [IPTG]. (a)** Western blot of HT-PBP2B GFP-FtsZ Δhag (strain SH147; Supplementary Table 1) and Δhag (strain SH211). Cultures were grown in rich media at 37°C with varying [IPTG] to induce HT-PBP2B expression. 5 µg of total protein from lysate was blotted. Spo0J was used as a loading control on a separate blot. The membranes were incubated with polyclonal antibodies specific for PBP2B or Spo0J. Bands were visualised by a chemiluminescence system. **(b)** Protein expression levels of HT-PBP2B across [IPTG] based on the normalized integrated intensities of bands in the Western blot. Sample sizes are listed in Supplementary Table 6. Uncropped images are provided as Supplementary Figure 12.

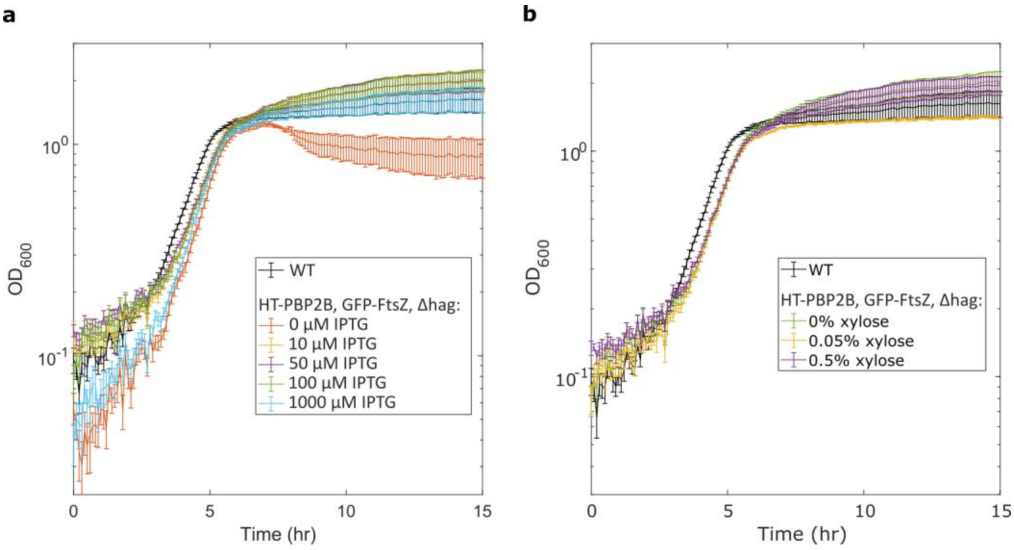


**Supplementary Figure 3. Effect of HT-PBP2B and GFP-FtsZ induction on growth in liquid culture.** Growth of HT-PBP2B GFP-FtsZ Δhag cultures (strain SH147) was monitored for 15 hours using a FLUOStar OPTIMA plate reader (BMG Labtech) and compared to that of wild-type (PY79) (Supplementary Table 1). Both strains were grown in LB at 30°C. Mean values ± SD of triplicate repeats are plotted. **(a)** HT-PBP2B expression was induced at the level specified by the [IPTG] shown, while GFP-FtsZ was not induced (0% xylose). **(b)** GFP-FtsZ expression was induced at the level specified by the [xylose] shown, while HT-PBP2B expression was induced with a constant 100 µM IPTG.

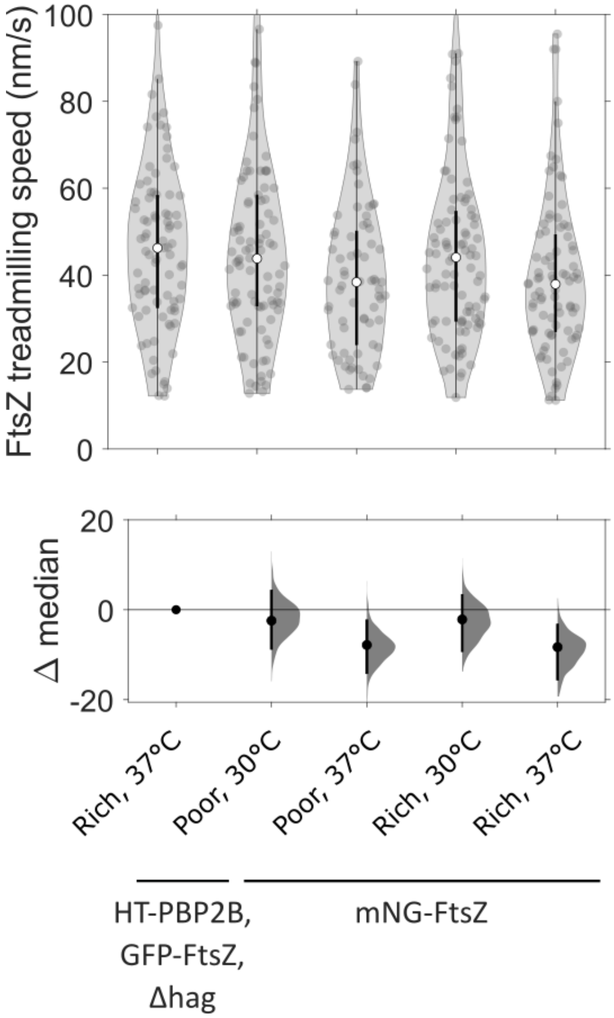


**Supplementary Figure 4. Speed of FtsZ treadmilling in HT-PBP2B, GFP-FtsZ, Δhag strain compared to previous measurements.** FtsZ treadmilling speeds were measured by TIRF microscopy using a HT-PBP2B, GFP-FtsZ, Δhag strain (strain SH147; Supplementary Table 1) and compared to treadmilling speeds in a strain expressing mNeonGreen-FtsZ (mNG-FtsZ) from an IPTG-inducible promoter (strain bWM4; Supplementary Table 1) measured across different growth conditions (poor vs. rich media, 30°C vs. 37 °C). HT-PBP2B, GFP-FtsZ, Δhag cultures were grown in rich media (PHMM) at 37°C with 0.075% xylose to induce a low level of GFP-FtsZ expression. Treadmilling speeds were determined by manually tracing filament trajectories on kymographs, as done previously[1]. Data for all mNG-FtsZ conditions are taken from Whitley et al[1]. Violin plots: white circles, median; thick black lines, interquartile range; thin black lines, 1.5x interquartile range. DABEST plots: black circle, median difference between indicated conditions; black lines, 95% confidence interval of median difference. Sample sizes are listed in Supplementary Table 6.

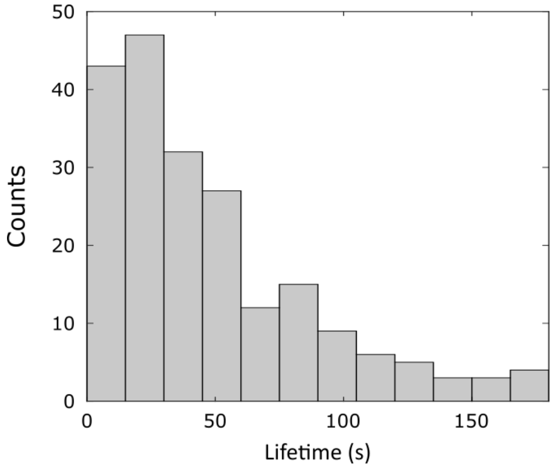


**Supplementary Figure 5. Lifetime of immobile HT-PBP2B tracks.** Histogram of lifetimes for immobile track segments from HT-PBP2B GFP-FtsZ Δhag cells (strain SH147) grown in PHMM at 30°C. Here, immobile is defined as a track segment with speed less than 4 nm/s. Sample sizes are listed in Supplementary Table 6.

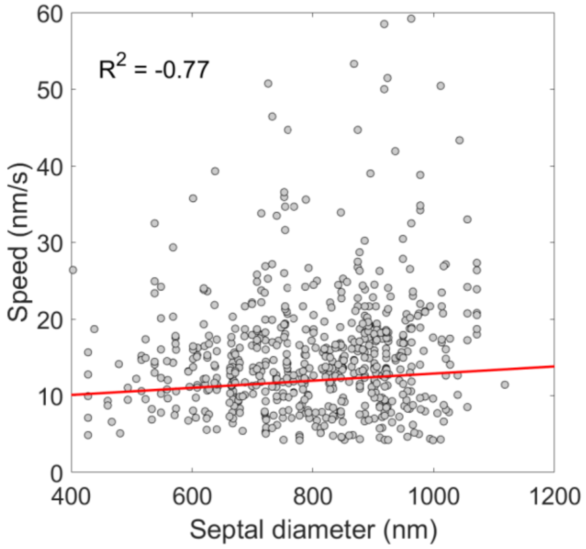


**Supplementary Figure 6. Correlation between HT-PBP2B speed and septal diameter.** Scatter plots showing the speeds of processive HT-PBP2B track segments (speeds between 4 and 60 nm/s) and the associated diameters of the septa around which they were moving under our standard conditions (rich media, 30°C).

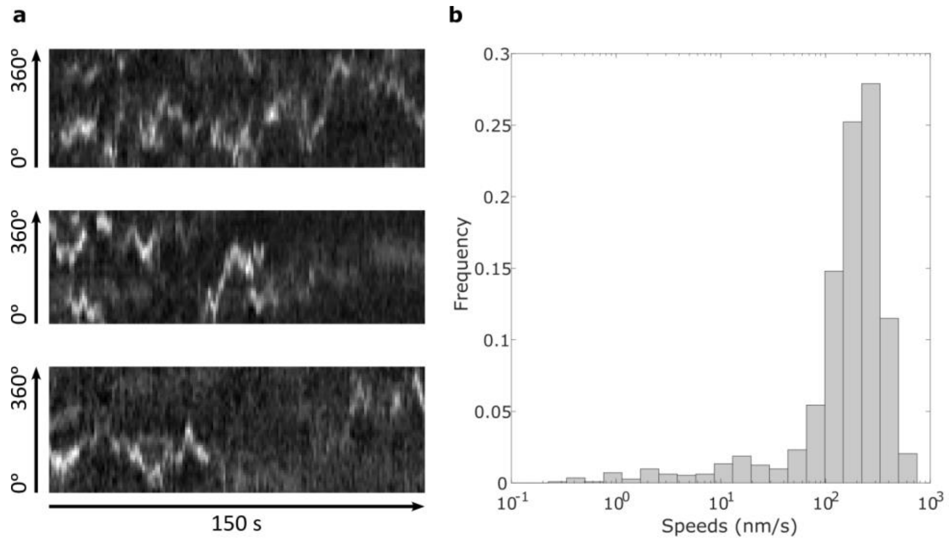


**Supplementary Figure 7. Motion of HT-PBP2B molecules outside the septal ring area. (a)** Example radial kymographs of HT-PBP2B (rich media, 30°C) showing motion outside the septal ring area (*i.e.* at a random section of the cell sidewall). **(b)** Histogram of HT-PBP2B speeds for molecules observed outside the septal ring area plotted on logarithmic x axis, measured from linear segments on kymographs.

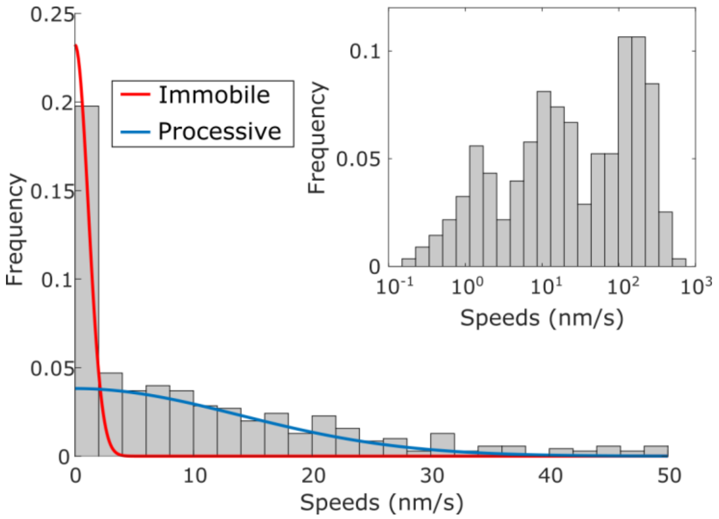


**Supplementary Figure 8. Speeds of HT-PBP2B molecules with high inducer concentration.** Histogram of HT-PBP2B speeds in HT-PBP2B GFP-FtsZ Δhag cells (strain SH147; Supplementary Table 1) grown in rich media with 1 mM IPTG and 0.075% xylose at 30°C with 250-500 pM JFX554 HaloTag ligand and imaged using VerCINI. Red and blue lines show fits to the data. *Inset*: Histogram of speeds plotted on logarithmic x axis, showing three populations.

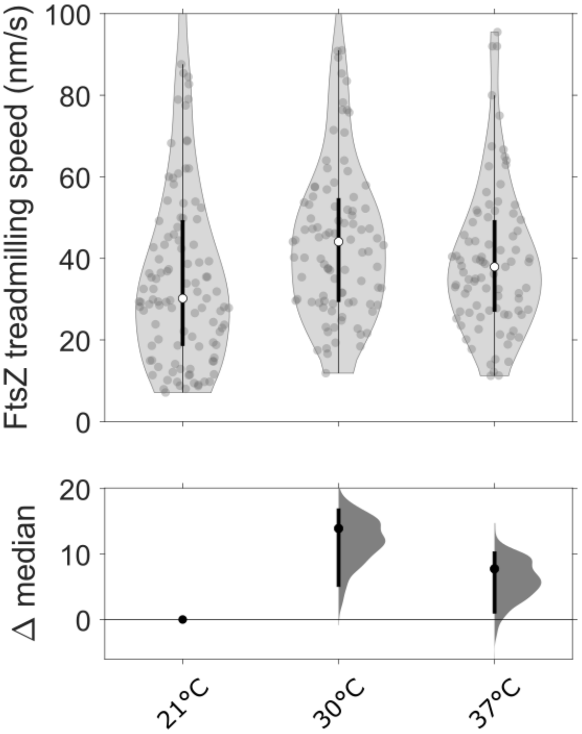


**Supplementary Figure 9. Effect of temperature on FtsZ treadmilling speed.** FtsZ treadmilling speeds were measured by TIRF microscopy using a strain expressing mNeonGreen-FtsZ from an IPTG-inducible promoter (strain bWM4; Supplementary Table 1) grown in rich media at 21°C and compared to previous measurements of the same strain in the same media under varying temperatures. Cultures were incubated with 25 µM IPTG 1 hr prior to imaging to induce mNeonGreen-FtsZ expression. Treadmilling speeds were determined by manually tracing filament trajectories on kymographs, as done previously[1]. Data for 30°C and 37°C are taken from Whitley et al. 2021[1]. Violin plots: white circles, median; thick black lines, interquartile range; thin black lines, 1.5x interquartile range. DABEST plots: black circle, median difference between indicated conditions; black lines, 95% confidence interval of median difference. Sample sizes are listed in Supplementary Table 6.

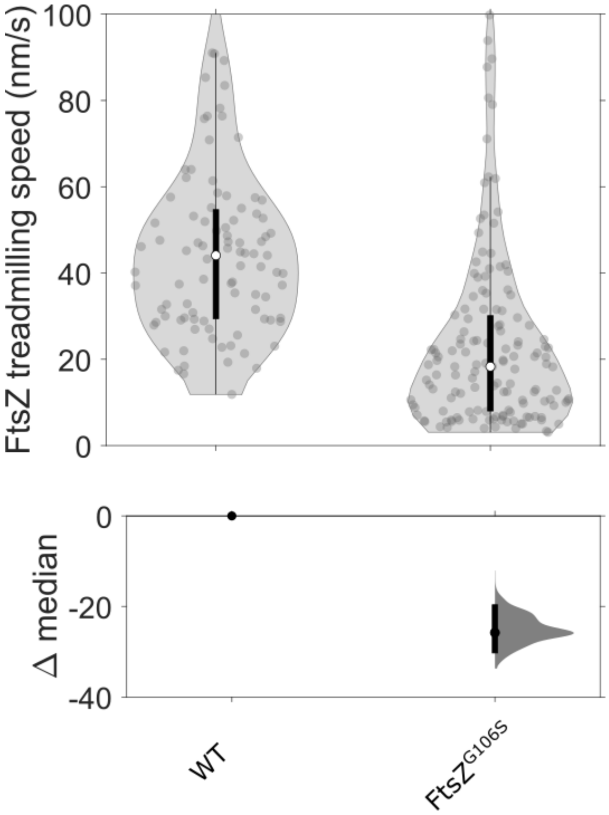


**Supplementary Figure 10. Effect of FtsZ$^{G106S}$ mutation on treadmilling speed.** FtsZ treadmilling speeds were measured for cells expressing FtsZ$^{G106S}$ (strain SH203; Supplementary Table 1) in rich media at 30°C and compared to treadmilling speeds in a strain expressing mNeonGreen-FtsZ from an IPTG-inducible promoter (strain bWM4) measured under the same conditions. FtsZ$^{G106S}$ cells were incubated with 0.075% xylose to induce GFP-FtsZ expression. Treadmilling speeds were determined by manually tracing filament trajectories on kymographs, as done previously[1]. Data for WT are taken from Whitley et al. 2021[1]. Violin plots: white circles, median; thick black lines, interquartile range; thin black lines, 1.5x interquartile range. DABEST plots: black circle, median difference between indicated conditions; black lines, 95% confidence interval of median difference. Sample sizes are listed in Supplementary Table 6.

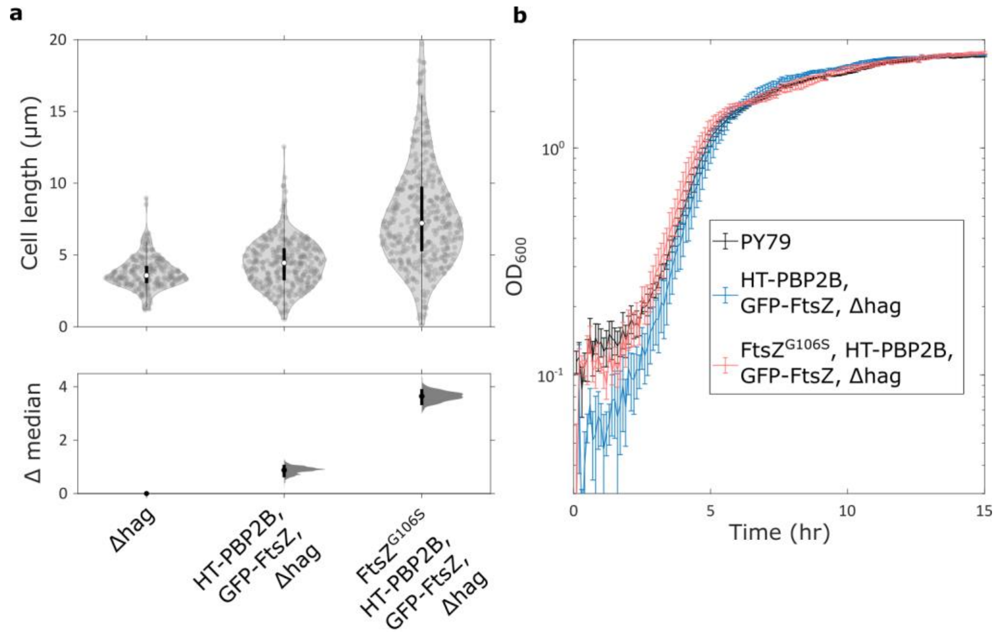


**Supplementary Figure 11. Effect of FtsZ$^{G106S}$ mutation on cell lengths and growth in liquid culture.** **(a)** Lengths of FtsZ$^{G106S}$ HT-PBP2B GFP-FtsZ Δhag cells (strain SH203) were measured by microscopy of Nile Red-stained cells and compared to those of HT-PBP2B GFP-FtsZ Δhag and Δhag cells (strains SH147 and SH211, respectively). All strains were grown in PHMM at 30°C. HT-PBP2B expression was induced with 100 µM IPTG in both strains containing HT-PBP2B, while GFP-FtsZ was not induced in either case (0% xylose). Violin plots: white circles, median; thick black lines, interquartile range; thin black lines, 1.5x interquartile range. DABEST plots: black circle, median difference between indicated conditions; black lines, 95% confidence interval of median difference. Sample sizes are listed in Supplementary Table 6. **(b)** Growth was monitored for 15 hours using a FLUOStar OPTIMA plate reader (BMG Labtech) at 30°C. Mean values ± SD of triplicate repeats are plotted. All strains were grown in LB at 30°C. HT-PBP2B expression was induced with 100 µM IPTG in both strains containing HT-PBP2B, while GFP-FtsZ was not induced in either case (0% xylose).

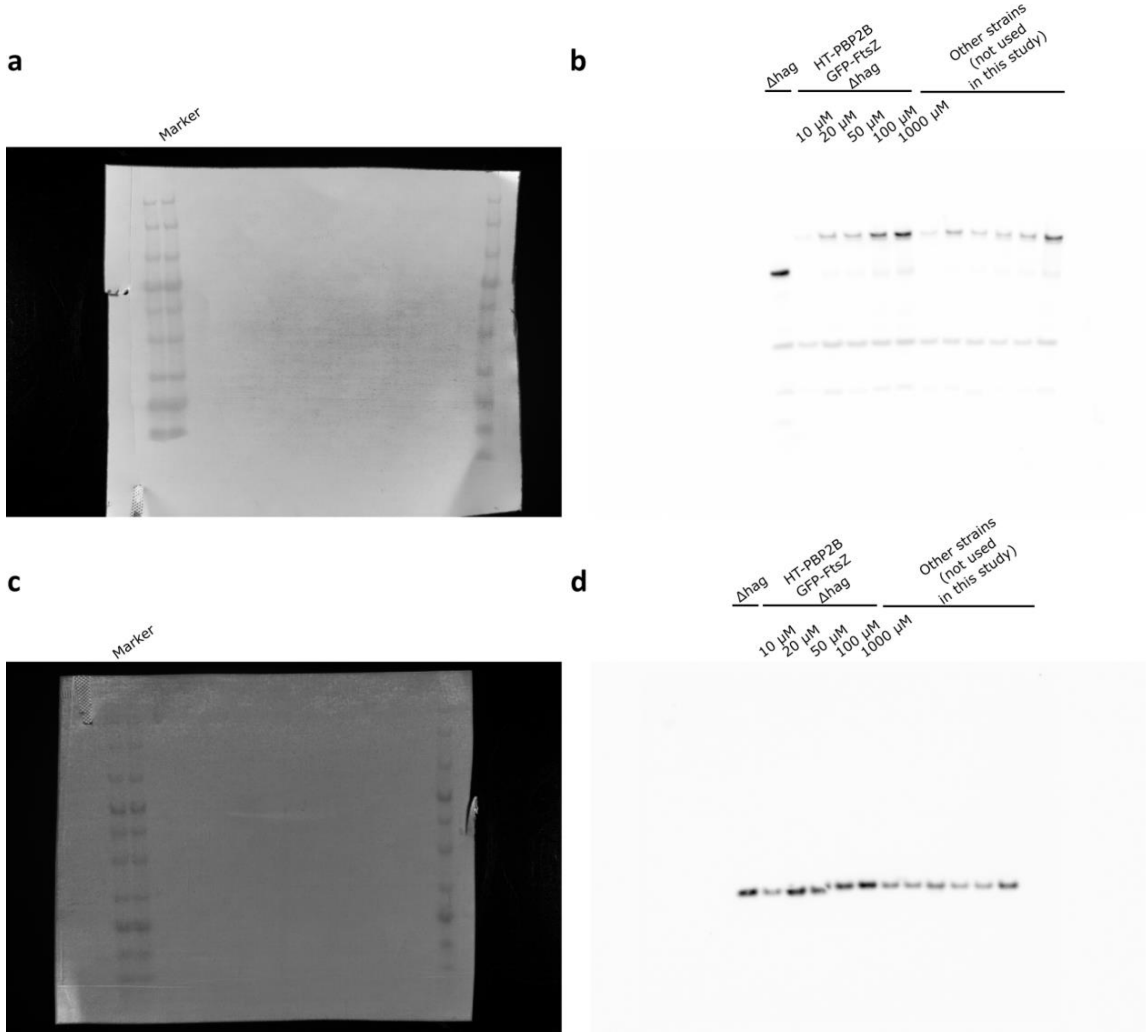


**Supplementary Figure 12. Uncropped images of anti-PBP2B and anti-Spo0J Western blots.** Uncropped Western blot images of HT-PBP2B GFP-FtsZ Δhag (strain SH147; Supplementary Table 1) and Δhag (strain SH211), corresponding to cropped images in Supplementary Figure 2. **(a)** White light illumination of anti-PBP2B blot. **(b)** Chemiluminescence of anti-PBP2B blot. **(c)** White light illumination of anti-Spo0J blot. **(d)** Chemiluminescence of anti-Spo0J blot.

# REFERENCES

1. Whitley, K. D. *et al.* FtsZ treadmilling is essential for Z-ring condensation and septal constriction initiation in Bacillus subtilis cell division. *Nat. Commun.* **12**, 2448 (2021).
