# Supplementary material for: Peptidoglycan synthesis drives a single population of septal cell wall synthases during division in Bacillus subtilis
Source: Nat Microbiol. 2024 Mar 13;9(4):1064–74. doi: 10.1038/s41564-024-01650-9 (PMC10994842; doi:10.1038/s41564-024-01650-9)
Supplement: Supplementary file 2 — Reporting Summary [file 41564_2024_1650_MOESM2_ESM.pdf]

nature portfolio

Corresponding author(s): Kevin D. Whitley and Séamus Holden

Last updated by author(s): Feb 20, 2024

Reporting Summary

Nature Portfolio wishes to improve the reproducibility of the work that we publish. This form provides structure for consistency and transparency in reporting. For further information on Nature Portfolio policies, see our Editorial Policies and the Editorial Policy Checklist.

Statistics

For all statistical analyses, confirm that the following items are present in the figure legend, table legend, main text, or Methods section.

n/a	Confirmed	
☐	☒	The exact sample size (*n*) for each experimental group/condition, given as a discrete number and unit of measurement
☐	☒	A statement on whether measurements were taken from distinct samples or whether the same sample was measured repeatedly
☐	☒	The statistical test(s) used AND whether they are one- or two-sided<br>*Only common tests should be described solely by name; describe more complex techniques in the Methods section.*
☒	☐	A description of all covariates tested
☐	☒	A description of any assumptions or corrections, such as tests of normality and adjustment for multiple comparisons
☐	☒	A full description of the statistical parameters including central tendency (e.g. means) or other basic estimates (e.g. regression coefficient) AND variation (e.g. standard deviation) or associated estimates of uncertainty (e.g. confidence intervals)
☐	☒	For null hypothesis testing, the test statistic (e.g. *F*, *t*, *r*) with confidence intervals, effect sizes, degrees of freedom and *P* value noted<br>*Give P values as exact values whenever suitable.*
☒	☐	For Bayesian analysis, information on the choice of priors and Markov chain Monte Carlo settings
☒	☐	For hierarchical and complex designs, identification of the appropriate level for tests and full reporting of outcomes
☒	☐	Estimates of effect sizes (e.g. Cohen's *d*, Pearson's *r*), indicating how they were calculated

Our web collection on statistics for biologists contains articles on many of the points above.

Software and code

Policy information about availability of computer code

Data collection	Data collected using either Micro-Manager (v2.0gamma), NS-Elements (v5.42.02), or Zen Black 2.3 (v16.0.14.316).
Data analysis	Videos analysed using Fiji (v1.54) with open-source plugins PureDenoise-GPU (v0.1.0) and PureDenoise-CPU (v0.1.0) (https://github.com/ZikaiSun/PureGpu/tree/main).<br><br>Extended Data Figures 4 and 6, along with Supplementary Videos 14-16 show data from molecules manually tracked using TrackMate (v7.10.2).<br><br>Further data analysis done using Matlab with custom code available on the Whitley lab Github page: https://github.com/WhitleyLab/Vercini_spt_analysis<br><br>The divisome complex was modelled by AlphaFold2, using ColabFold (v1.3.0) and AlphaFold-Multimer (v2).

For manuscripts utilizing custom algorithms or software that are central to the research but not yet described in published literature, software must be made available to editors and reviewers. We strongly encourage code deposition in a community repository (e.g. GitHub). See the Nature Portfolio guidelines for submitting code & software for further information.

Data

Policy information about availability of data

All manuscripts must include a data availability statement. This statement should provide the following information, where applicable:

- Accession codes, unique identifiers, or web links for publicly available datasets
- A description of any restrictions on data availability
- For clinical datasets or third party data, please ensure that the statement adheres to our policy

All source data for figures and results in this paper can be found in the Figshare repository: https://doi.org/10.25405/data.ncl.c.7078312

The sequences for performing protein structure predictions were downloaded from the UniProtKB database (Q07868 (PBP2B); Q07867 (FtsL); O07639 (FtsW); P16655 (DivIB); P37471 (DivIC)).

Research involving human participants, their data, or biological material

Policy information about studies with human participants or human data. See also policy information about sex, gender (identity/presentation), and sexual orientation and race, ethnicity and racism.

Reporting on sex and gender	N/A
Reporting on race, ethnicity, or other socially relevant groupings	N/A
Population characteristics	N/A
Recruitment	N/A
Ethics oversight	N/A

Note that full information on the approval of the study protocol must also be provided in the manuscript.

Field-specific reporting

Please select the one below that is the best fit for your research. If you are not sure, read the appropriate sections before making your selection.

☒ Life sciences ☐ Behavioural & social sciences ☐ Ecological, evolutionary & environmental sciences

For a reference copy of the document with all sections, see nature.com/documents/nr-reporting-summary-flat.pdf

Life sciences study design

All studies must disclose on these points even when the disclosure is negative.

Sample size	No a priori sample size calculations were performed. No specific sample size was chosen (exceptions noted below) as the single cell/ single molecule nature of the measurements means moderate to large sample size, sufficient for robust statistical analysis, is usually straightforward to achieve. N>80 for each experiment or analysis in the paper, which was sufficient to evaluate results. Where the data were suitable, sample data violin plots/ histograms were evaluated post-hoc to check that the probability density function of the underlying distribution appeared well sampled; we observed that this was the case for all measurements. Numbers of cells, track segments, and other data points are all listed in Supplementary Table 6.<br>For bulk bacterial growth curves (Supplementary Figures 3 and 11b), three samples were prepared in order to estimate the variance of the measurement.
Data exclusions	No data was excluded.
Replication	The number of independent biological replicates for each experiment, defined as the number of experiments done using distinct and independently-prepared samples, can be found in Supplementary Table 6.
Randomization	Allocating experimental groups was not relevant for this study, as all bacterial cells of a particular strain are genetic clones.
Blinding	Blinding was neither possible nor necessary for this study, as 1) all bacterial cells of a particular strain are genetic clones and 2) analyses were not sufficiently subjective to require researcher blinding.

Reporting for specific materials, systems and methods

We require information from authors about some types of materials, experimental systems and methods used in many studies. Here, indicate whether each material, system or method listed is relevant to your study. If you are not sure if a list item applies to your research, read the appropriate section before selecting a response.

Materials & experimental systems

n/a	Involved in the study
☐	☒ Antibodies
☒	☐ Eukaryotic cell lines
☒	☐ Palaeontology and archaeology
☒	☐ Animals and other organisms
☒	☐ Clinical data
☒	☐ Dual use research of concern
☒	☐ Plants

Methods

n/a	Involved in the study
☒	☐ ChIP-seq
☒	☐ Flow cytometry
☒	☐ MRI-based neuroimaging

Antibodies

Antibodies used

Anti-Pbp2B, Merck, Cat. No. ABS2199
Anti-Spo0J, gift from Jeffery Errington lab
HRP-conjugated Anti-Rabbit IgG, Merck, Cat. No. A8275

Validation

Anti-Pbp2B (from Merck website):
Application Anti-Pbp2B, Cat. No. ABS2199, is a rabbit polyclonal antibody that detects Penicillin-binding protein 2B (Pbp2B) and has been tested for use in immunofluorescence and Western Blotting.
Western Blotting Analysis: A 1:10,000 dilution from a representative lot detected Pbp2B in WT Bacillus Subtilis and GFP-Pbp2B (Courtesy of Dr. Richard Daniel at Newcastle University, UK).
Western Blotting Analysis: A 1:10,000 dilution from a representative lot detected His-PbpB recombinant protein (Courtesy of Dr. Richard Daniel at Newcastle University, UK).
Immunofluorescence Analysis: A representative lot detected Pbp2B in immunofluorescence applications (Daniel, R.A., et. al. (2000). Mol Microbiol. 35(2):299-311).
Western Blotting Analysis: A representative lot detected Pbp2B in Western Blotting applications (Bisson-Filho, A.W., et. al. (2017). Science. 355(6326):739-743; Adams, D.W., et. al. (2016). Mol Microbiol. 99(6):1028-42).

Anti-Spo0J (non-commercial):
Anti-Spo0J is a rabbit polyclonal antibody that detects Spo0J and has been tested for use in immunofluorescence and Western blotting: Glaser, P. & Sharpe, M.E. et al. (1997). Genes and Development. 11(9):1160-8.

Anti-Rabbit IgG (from Merck website):
Co-immunoprecipation and western blot analysis of C33A cell lysates were performed using HRP conjugated goat anti-rabbit IgG as the secondary antibody.
Immunohistochemistry was performed on frozen sections (10um) of mouse intestine, liver, and spleen using HRP-conjugated goat anti-rabbit IgG as the secondary antibody. Prior to incubation with the secondary, sections were treated with a mixture of MeOH/hydrogen peroxide 30% to block endogenouse peroxidases.
Prepared using the periodate method described by Wilson, M.B., and Nakane, P.K., in Immunofluorescence and Related Staining Techniques, Elsevier/North Holland Biomedical Press, Amsterdam, p215 (1978).
